# Genome-wide identification and expression profiling of the ABI5 gene family in foxtail millet (*Setaria italica*)

**DOI:** 10.1186/s12870-024-04865-4

**Published:** 2024-03-02

**Authors:** Yinyuan Wen, Zeya Zhao, Liuna Cheng, Shixue Zhou, Mengyao An, Juan Zhao, Shuqi Dong, Xiangyang Yuan, Meiqiang Yin

**Affiliations:** College of Agronomy, Shanxi Agricultural University, Taigu, 030801 China

**Keywords:** Foxtail millet, SiABI5, Phylogenetic analysis, Expression analysis, Abiotic stress

## Abstract

**Background:**

ABA Insensitive 5 (ABI5) is a basic leucine zipper transcription factor that crucially influences plant growth, development, and stress response. However, there is minimal research on the *ABI5* family in foxtail millet.

**Results:**

In this study, 16 *ABI5* genes were identified in foxtail millet, and their sequence composition, gene structures, cis-acting elements, chromosome positions, and gene replication events were analyzed. To more thoroughly evaluate the developmental mechanisms of the *SiABI5* family during evolution, we selected three dicotyledons (*S. lycopersicum*, *A. thaliana*, *F. tataricum*) and three (*Z. mays*, *O. sativa*, *S. bicolor*) specific representative monocotyledons associated with foxtail millet for comparative homology mapping. The results showed that foxtail millet *ABI5* genes had the best homology with maize. A promoter sequence analysis showed that the SiABI5s contain numerous cis-acting elements related to hormone and stress responses, indicating that the regulation of *SiABI5* expression was complex. The expression responses of 16 genes in different tissues, seed germination, and ear development were analyzed. A total of six representative genes were targeted from five subfamilies to characterize their gene expression responses to four different abiotic stresses. Overexpression of *SiABI5.12* confers tolerance to osmotic stress in transgenic Arabidopsis thaliana, which demonstrated the function of SiABI5 responded to abiotic stress.

**Conclusions:**

In summary, our research results comprehensively characterized the *SiABI5* family and can provide a valuable reference for demonstrating the role of *SiABI5s* in regulating abiotic stress responses in foxtail millet.

**Supplementary Information:**

The online version contains supplementary material available at 10.1186/s12870-024-04865-4.

## Introduction

Foxtail millet (*Setaria italica*) is one of the oldest cultivated crops in the world, as it was domesticated from the wild relatives of green foxtail in northern China over 10,000 years ago [1, 2]. *S. italica* has many beneficial traits, such as tolerance to abiotic stress (e.g., drought, heat, and low soil nutrients) and high photosynthetic efficiency [3, 4]. More importantly, *S. italica* benefits from a small diploid genome [5–7], self-pollination, and multiple single-spike seed settings, which facilitate its analysis and modification in both fundamental and applied research [8].

Plant transcription factors (TFs) perform biological functions by regulating the transcription of target genes [9]. These TFs play important roles in plants, including in regulating flower growth [10, 11], carbon and nitrogen metabolism [12], the Circadian rhythm [13], cell differentiation [14], hormone reaction [15], and resistance to disease [16]. The basic leucine zipper (bZIP) family is the most extensive transcription factor family and is named and characterized according to its conserved bZIP domain [17]. Abscisic Acid INSENSITIVE 5 (ABI5) is a bZIP transcription factor [18] comprising four conserved domains from C1 to C4 and one bZIP domain [19], with the former containing serine residues (S42, S145, and S439) and threonine residues (T201) and the latter containing lysine residues (K344 and K391) related to ubiquitination and susceptibility [20].

Plant ABI5 is an important transcription factor in the abscisic acid (ABA) signaling pathway [21], as it influences biological processes such as seed development, plant growth, and anthocyanin accumulation, as well as the responses of plants to stressors such as drought and low nitrogen [22]. For example, *ABI5* plays an essential role in repressing seedling growth under osmotic, salt, and cold stresses in *Arabidopsis* [23]. In *Arabidopsis thaliana*, *abi5* identified using inhibition screening reduced plant susceptibility to ABA and inhibited seed germination [24]. In maize, the *ABI5* expression is induced by a wide spectrum of stressors, including abscisic acid, salicylic acid, NaCl, high and low temperatures, wounding, and mannitol treatment [25]. In barley, the transcription factor ABI5 plays a crucial role in regulating various processes associated with drought tolerance. These include membrane protection, the accumulation of flavonoids, and stomatal closure. *ABI5* fulfills these functions through activating the expression of stress-responsive genes, thus aiding in the plant’s adaptation to drought stress [26]. In rice, *ABI5* expressed under ABA and high-salt conditions is downregulated under drought and low-temperature conditions. Moreover, as a transcription factor, ABI5 can regulate stress and fertility in rice [27, 28]. Furthermore, *ABI5* interacts with other plant hormone signaling pathways to regulate seed germination [29]. For example, the jasmonate-ZIM domain is a negative regulator of jasmonic acid signaling that inhibits the transcriptional activity of *ABI5* and regulates wheat seed germination [30].

Thus, *ABI5* crucially influences plant growth, development, and stress responses. However, no previous research has incorporated the systematic identification, classification, evolution, and gene function analysis of the *ABI5* gene family in *S. italica*. Therefore, to address this research gap, we comprehensively analyzed the sequence composition, gene structures, cis-acting elements, chromosome positions, and gene replication events of 16 *ABI5* gene members in the *S. italica* genome (*SiABI5*). As the evolutionary relationship of *SiABI5* genes has previously been analyzed in several species, including rice, *Arabidopsis thaliana*, sorghum, maize, tomato, and Tartary buckwheat, we also investigated the grouping, motif composition, collinearity, and evolutionary relationships between *SiABI5* genes and those of other plants. We also determined the expression patterns of *ABI5* during foxtail millet germination and maturation. Our study objective was thus to reveal the roles of specific *ABI5* members in different biological processes in *S. italica*, as well as their expression under four types of abiotic stress. This study not only identifies important *ABI5* genes under growth, development, and stress treatments, but also provides insights for future research on the *ABI5* gene family in other plants.

## Results

### Identification of ABI5 proteins in foxtail millet

Sixteen full-length *SiABI5* genes were identified in *S. italica*, which were located on seven of the nine chromosomes; based on their positions, the genes were renamed *SiABI5.1* to *SiABI5.16*.

According to the physicochemical property assessments (Table S1), the lengths of the 16 SiABI5 proteins varied markedly. The smallest protein was SiABI5.14 (147 aa), with a molecular weight of 16.54 kDa, and the largest protein was SiABI5.13 (409 aa), with a molecular weight of 43.31 kDa. Most proteins ranged in length from 300 to 400 amino acids. Among the 16 SiABI5 proteins, the isoelectric points ranged from 5.32 (SiABI5.1) to 9.97 (SiABI5.14). The isoelectric point was greater than 7.00 for 11 proteins, indicating that more than 68% of SiABI5 proteins were basic proteins. The instability index ranged from 42.97 (SiABI5.15) to 73.99 (SiABI5.8), indicating that all genes were unstable. The aliphatic indices ranged from 62.49 (SiABI5.7) to 80.83 (SiABI5.11). The grand average of hydropathy values indicated that all 16 proteins were hydrophilic. A subcellular localization analysis predicted that six genes were located in the nucleus, nine genes were located in the chloroplasts, and one was located in the mitochondria.

### Multiple sequence alignment, phylogenetic analysis, and classification of SiABI5 genes

**Fig. 1:**
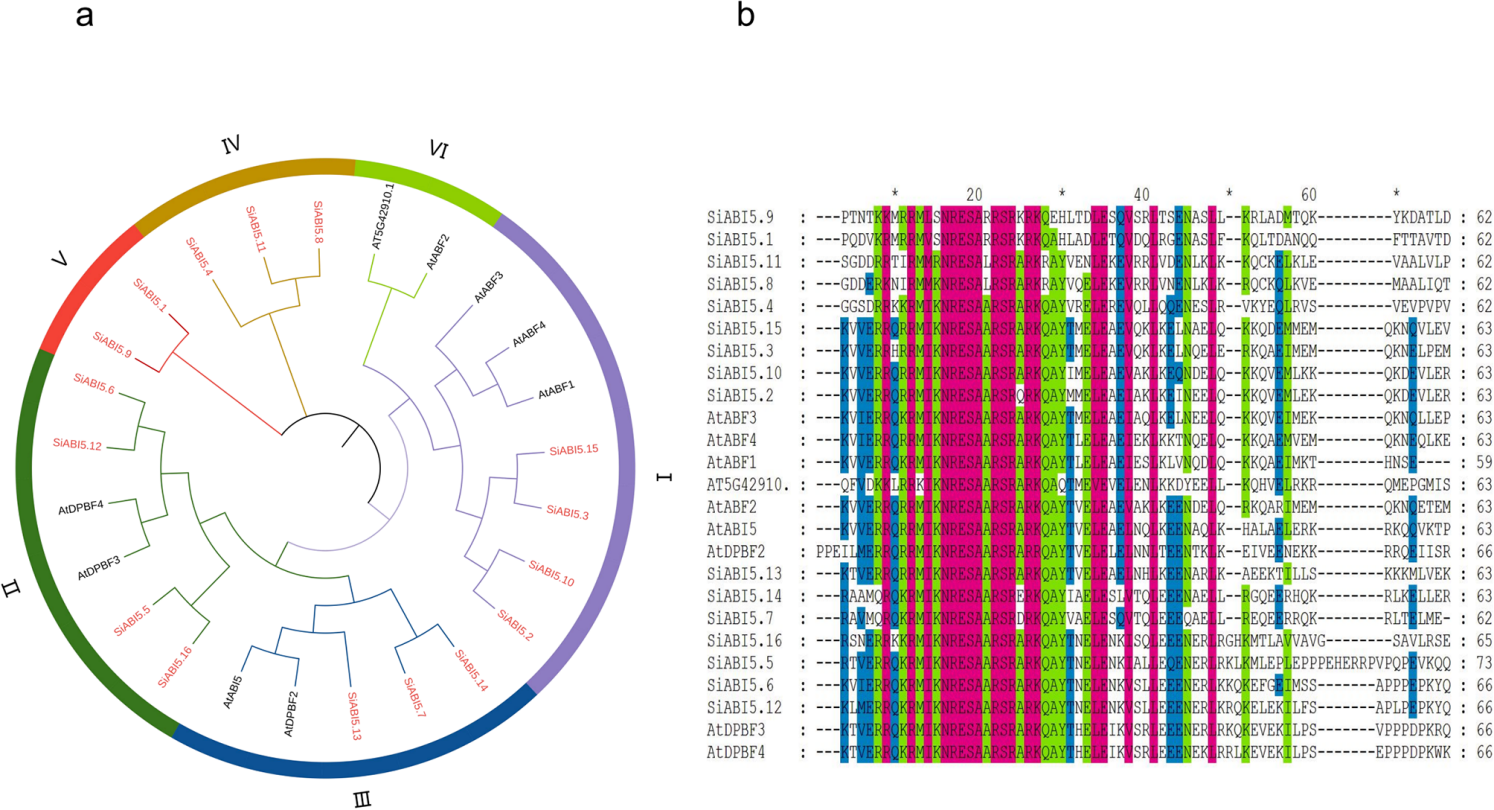
Phylogenetic tree and bZIP domain sequence alignment of *S. italica* ABI5 proteins. The phylogenetic tree was derived using the ML method using MEGA 11 and shows six phylogenetic subfamilies. (**a**) Phylogenetic tree of the relationship between *S. sitaria* and *A. thaliana* ABI5 proteins. (**b**) bZIP domain comparison

To explore the evolutionary relationship of *ABI5* genes in *S. italica*, nine *A. thaliana ABI5* subfamily genes and 16 *SiABI5* genes were used to construct a phylogenetic tree using MEGAX software version 11. According to the classification method of the *AtABI5* subgroup gene family, as shown in the evolutionary tree, the *ABI5* gene family was divided into six subgroups: I to VI (Fig. 1a). However, no *SiABI5* members were present in group VI, and the final *SiABI5* gene family was divided into five subfamilies. Among these subgroups, groups I and II contained the most *SiABI5* gene members, with four members each. Groups III and IV had three members, whereas group V had the fewest *SiABI5* gene members (two). According to the location of the bZIP domain, we extracted the bZIP domain sequence of *SiABI5* (approximately 65 amino acids) and used it for multiple sequence alignment. As shown in Fig. 1b, the foxtail millet *ABI5* domain contains highly conserved sequences such as RMI (RMV, RMM), NRESA, and RSR.

### Gene structure and motif composition of the SiABI5 gene family

**Fig. 2:**
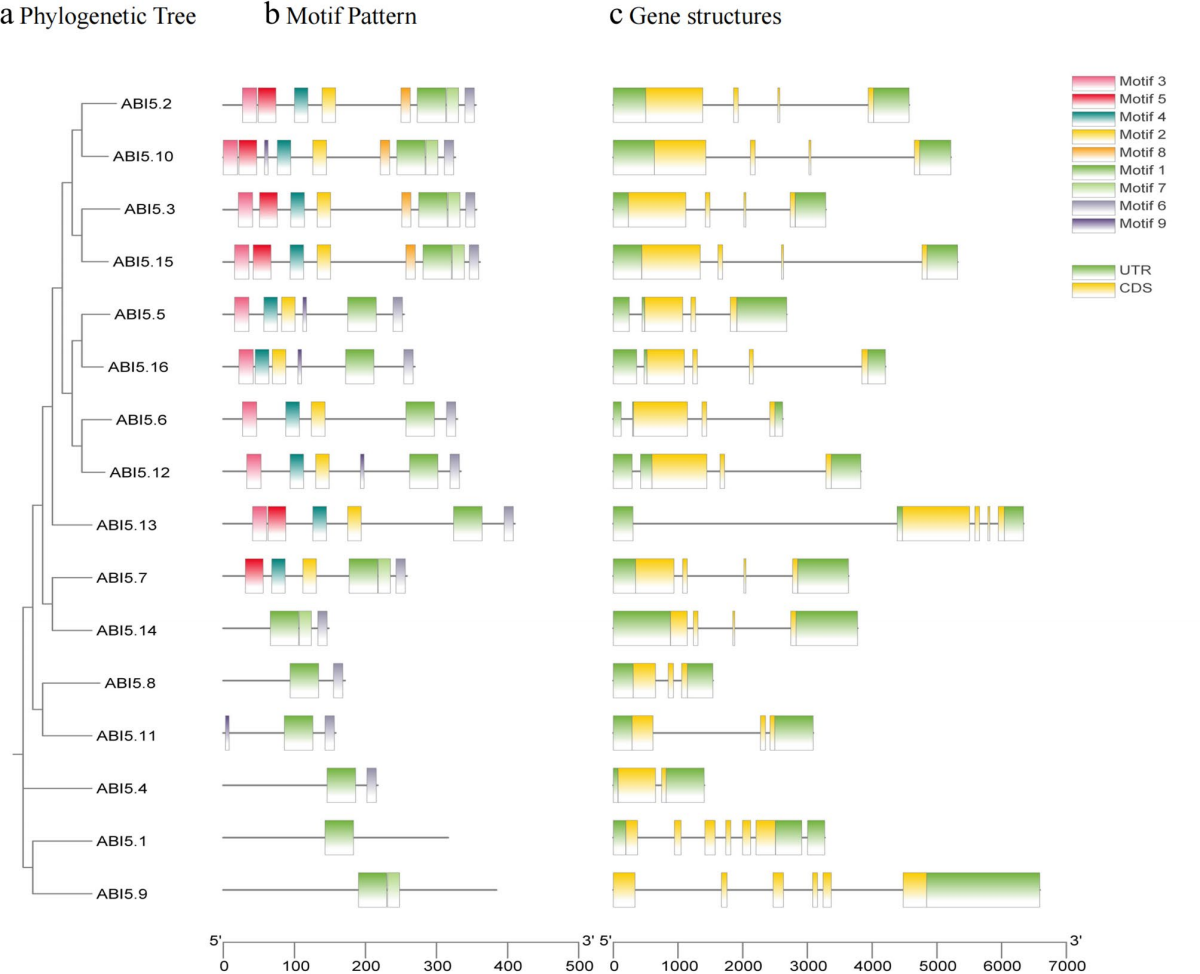
Phylogenetic relationships, conserved proteins, motif structures, and gene structures of 16 *SiABI5* genes from *S. italica*. (**a**) Phylogenetic tree was constructed based on the full-length sequences of SiABI5 proteins. (**b**) Amino acid motifs in SiABI5 proteins (1–9) represented by colored boxes. (**c**) Exon–intron structure of *SiABI5* genes. The green and yellow boxes represent the untranslated region and coding sequence, respectively. The gray lines represent introns

The evolution of the *ABI5* gene family in *S. italica* was further explored by studying the intron–exon structures of all *SiABI5* genes. As shown in Fig. 2, the intron–exon structures were similar within the same subfamily but showed substantial differences between different subfamilies. For example, subfamily V (*SiABI5.1*, *SiABI5.9*) had the largest number of introns, with an average intron number of 5.5. The other subfamilies contained only three or four introns. Subfamily IV, with the least number of introns, included *SiSABI5.11* and *SiABI5.8*, with two introns, and *SiABI5.4*, with only one intron. All *SiABI5* members exhibited a bZIP domain, but the *SiABI5.1* and *SiABI5.9* domains differed from the others.

To investigate variations in the conserved motifs of each SiABI5 protein, we utilized the MEME website to analyze the motif composition of the entire sequence, including the bZIP domain. The analysis revealed nine distinct conserved motifs, labeled as motifs 1–9 (Fig. 2b, Table S2). Motif 1 was distributed among all SiABI5 members, with ABI5.10 having the most motifs and ABI5.1 having the fewest motifs (one). In addition, all subfamilies contained motif 6, except subfamily V (SiABI5.1 and SiABI5.9). The motif arrangement was similar within the same subfamily, indicating that these protein structures were relatively conserved. This finding supports the reliability of the SiABI5 subfamily classification.

### Cis‑acting elements of the SiABI5 gene family

Further analysis of the promoter region 2,000 bp upstream of *SiABI5* identified 74 cis-acting elements (Table S3) that were classified into seven categories of elements: promoter-related, light-response, hormone-response, environmental stress-related, development-related, binding site-related, and other. Among these, 16 light-reaction-related elements and 12 development-related elements accounted for the largest proportion. Most *SiABI5* genes contained light-reaction-related elements (G-box, Sp1, and Box4-motif). Development-related elements, such as root-specific expression (as-1) and meristem expression elements (CAT-box), were also widely distributed in the *SiABI5* gene family. In addition, all *SiABI5* genes possessed cis-acting elements involved in abscisic acid (ABRE), and most *SiABI5* genes contained methyl jasmonate (TGACG-motif, CGTCA-motif), whereas a few genes also had cis-acting elements involved in auxin (TGA-element), gibberellin (P-box, GAREmotif), ethylene (ERE), and salicylic acid (TCA-element). Among the elements related to environmental stress, hypoxia-inducible (GC motif) and drought-inducible associated elements (MBS) and WRKY-type transcription factors (W-box) were widely distributed in the *SiABI5* genes. In all *SiABI5* genes, the core elements involved in transcription initiation (TATA and CAAT box) were consistently present among the promoter-associated elements, thus confirming the reliability of the promoter analysis. Finally, 21 other cis-acting elements were identified in the *SiABI5* genes; although STRE (AGGGG) and Unnamed_4 (CTCC) were found in all *SiABI5* genes, their exact function has not yet been discovered.

**Fig. 3:**
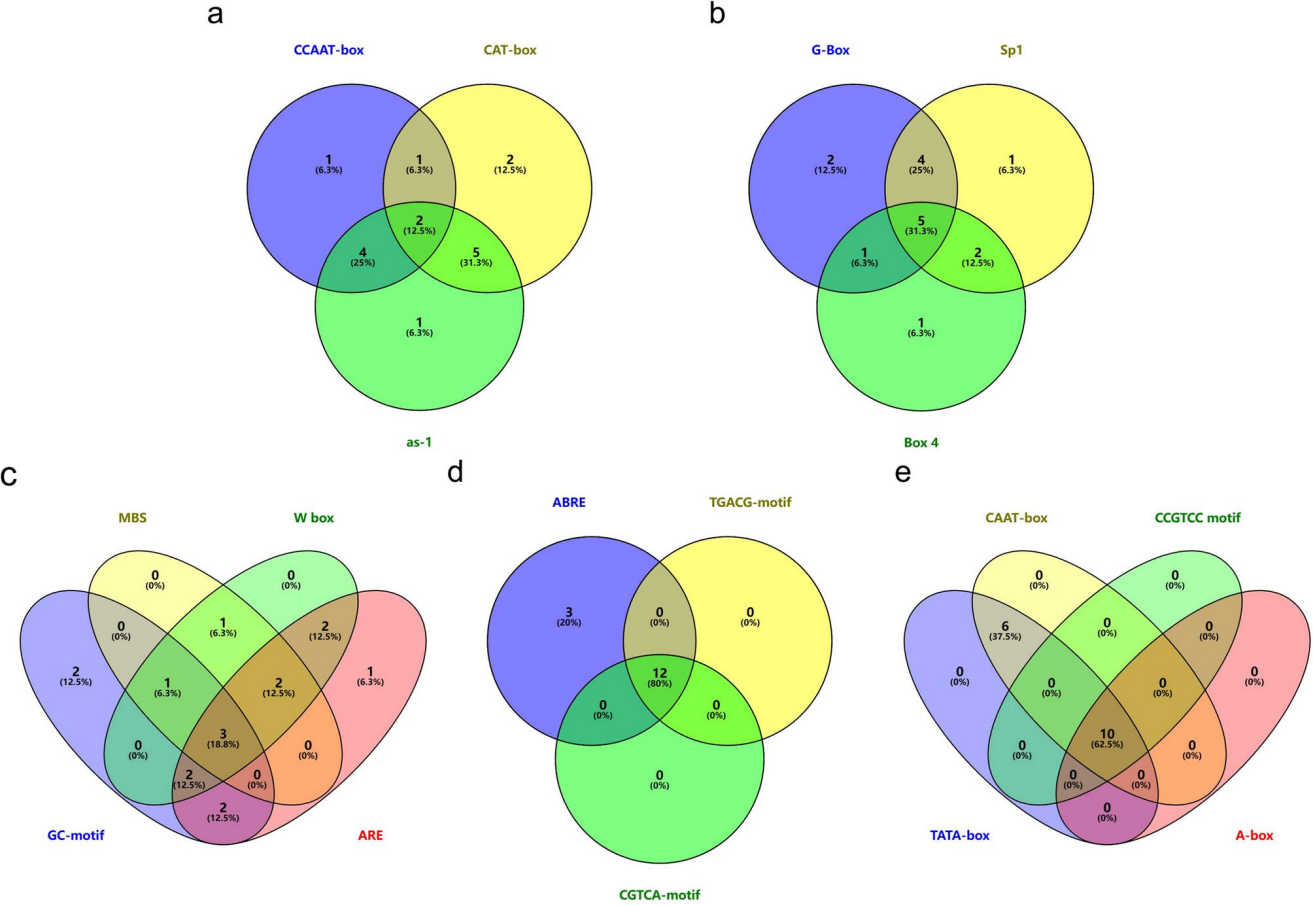
Cis-acting elements of *SiABI5* genes. (**a**) Development-related elements, (**b**) light-responsive elements, (**c**) environmental stress-related elements, (**d**) hormone-responsive elements, and (**e**) promoter-related elements

A Venn analysis was conducted to examine the importance of cis-acting elements observed in more than 10 genes (Fig. 3). For example, among the environmental stress-related elements, three *SiABI5* genes (*SiABI5.3*, *SiABI5.10*, and *SiABI5.14*) contained the ARE (AAACCA), GC-motif (CCCCCG), W-box (TTG ACC), and MBS (CAACTG) cis-acting elements. Among the light-response-related elements, five *SiABI5* genes (*SiABI5.1*, *SiABI5.9*, *SiABI5.11*, *SiABI5.12*, and *SiABI5.14*) simultaneously contained the G-Box, Sp1, and Box4 motif elements. Two *SiABI5* genes with development-related elements (*SiABI5.5* and *SiABI5.15*) simultaneously contained the as-1, CAT-box, and CCAAT-box. Twelve *SiABI5* genes with hormone-response elements (*SiABI5.2*, *SiABI5.3*, *SiABI5.4*, *SiABI5.5*, *SiABI5.6*, *SiABI5.7*, *SiABI5.8*, *SiABI5.9*, *SiABI5.11*, *SiABI5.13*, *SiABI5.15*, and *SiABI5.16*) contained the ABRE, TGACG-motif, and CGTCA-motif.

### Chromosomal distribution, gene duplication, and synteny analysis of SiABI5 genes

**Fig. 4:**
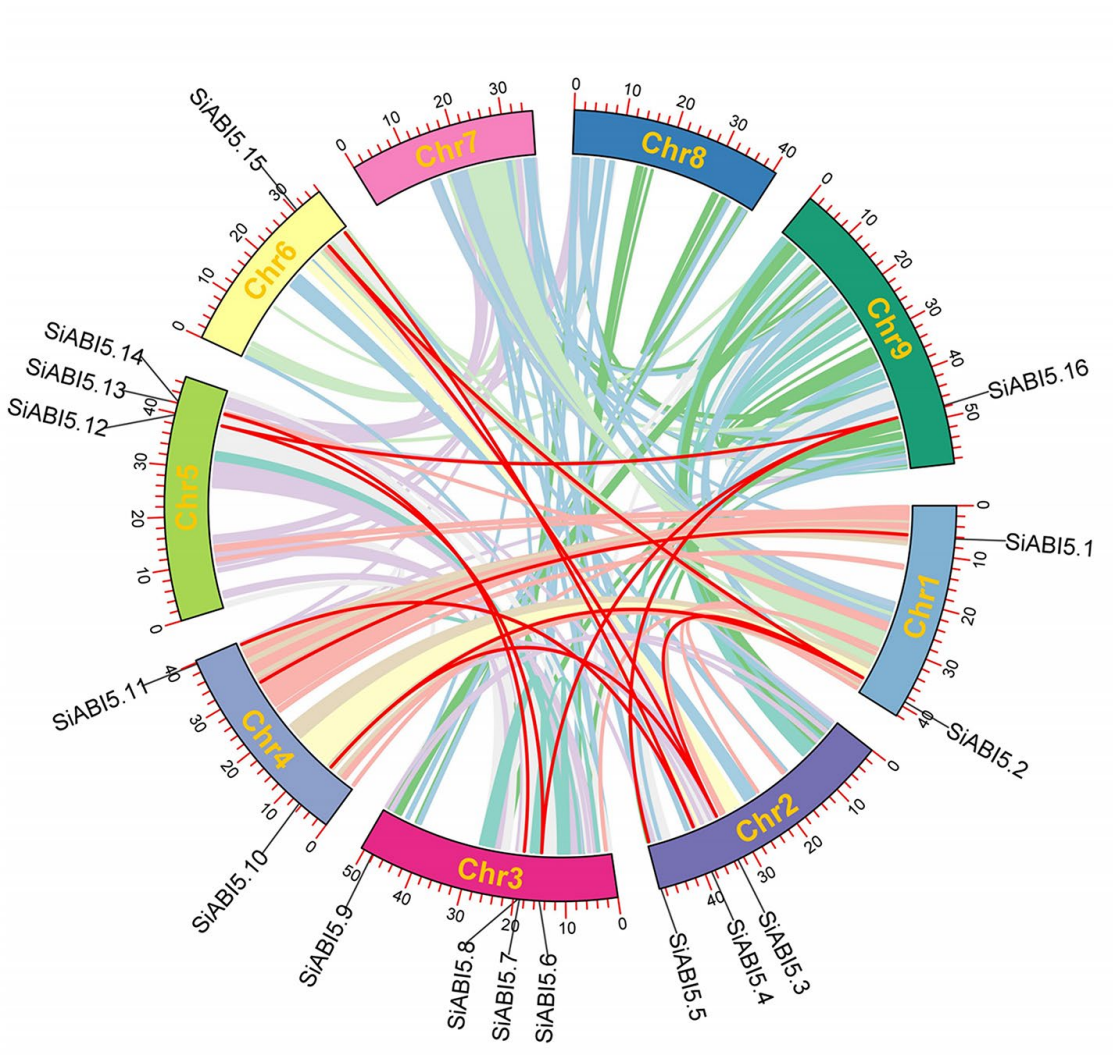
Schematic representation of the chromosomal distribution and synteny blocks of *S. italica ABI5* genes. Colored lines indicate all synteny blocks in the *S. italica* genome, and red lines indicate segmentally duplicated *ABI5* gene pairs. The chromosome number is indicated at the bottom of each chromosome

The distribution of 16 *SiABI5* genes on the nine chromosomes of foxtail millet was determined via chromosome mapping of the foxtail millet genome. *SiABI5* was not present on chromosomes VII and VIII, and the smallest number of genes was distributed on chromosomes VI and IX, which contained only one *SiABI5* gene. The remaining chromosomes harbored a total of two or three *SiABI5* genes each. Gene duplications are indispensable for the evolutionary process of gene families, as they contribute significantly to gene amplification and the emergence of novel functional genes. Consequently, we conducted a comprehensive analysis of the *SiABI5* gene’s duplication events across the foxtail millet genome. Through utilizing TBtools, we identified a total of 13 pairs of segmental duplication events within the *SiABI5* gene family (Fig. 4, Table S4). These segmental duplication genes were distributed on chromosomes 1/2/3, 4/5/6, and 9. These findings suggest that segmental duplication events contributed to the amplification and diversification of *SiABI5* during the evolution of the *SiABI5* gene family.

**Fig. 5:**
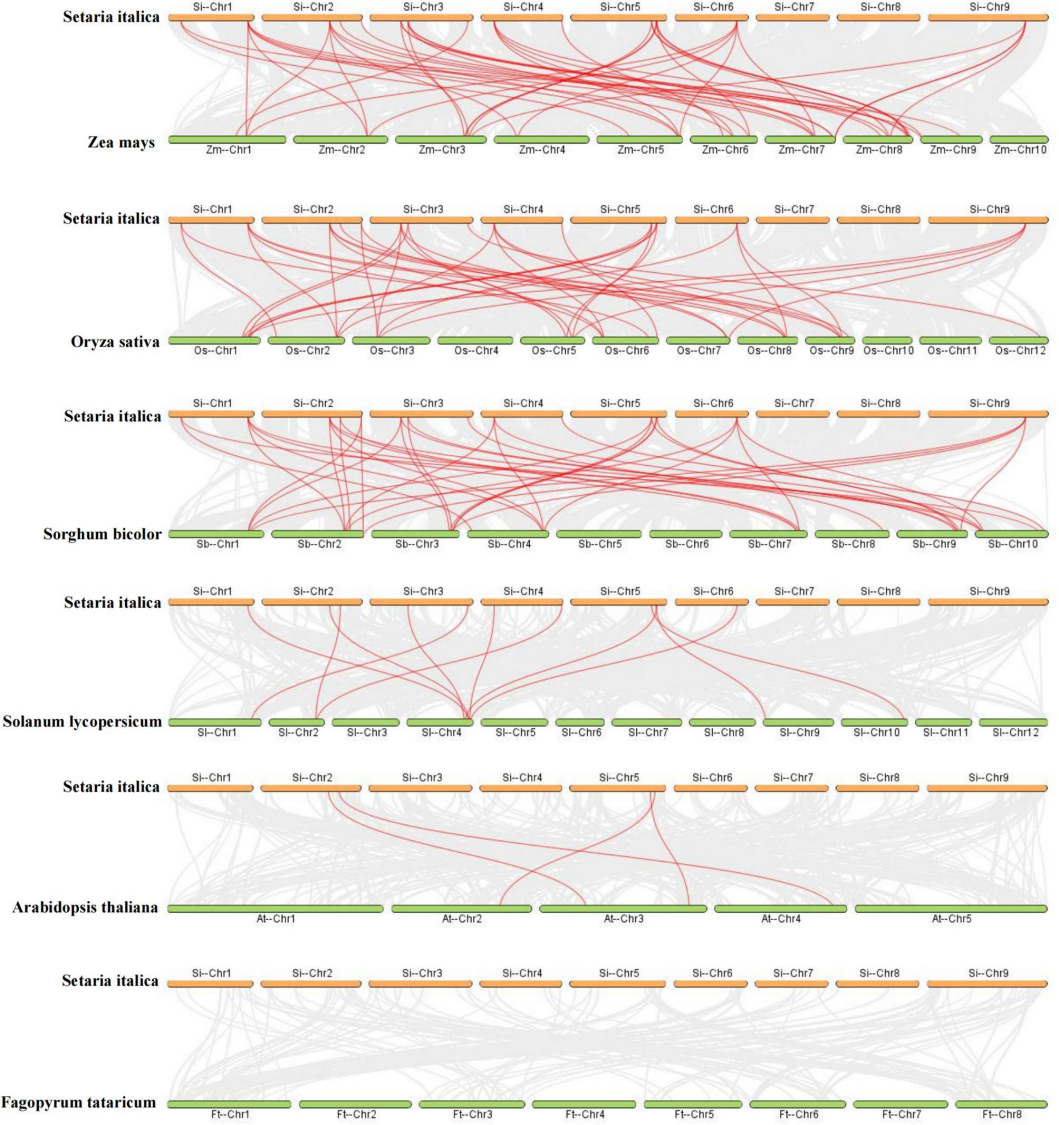
Synteny analysis of *ABI5* genes between *S. italica* and six representative plant species (*Z. mays, O. sativa, S. bicolor, S. lycopersicum, A. thaliana, F. tataricum*). Gray lines in the background indicate the collinear blocks within *S. italica* and other plant genomes, whereas red lines highlight syntenic *ABI5* gene pairs

To gain further insights into the phylogenetic mechanism of the *SiABI5* gene family, we constructed syntenic maps of six representative species of foxtail millet: three monocotyledons (rice, sorghum, and maize) and three dicotyledons (*Arabidopsis thaliana*, Tartary buckwheat, and tomato) (Fig. 5). Among the homologous genes of *SiABI5* and other species, those homologous to maize were the most common (45 pairs), followed by genes homologous to rice (38 pairs), sorghum (37 pairs), tomato (11 pairs), *Arabidopsis* (4 pairs), and Tartary buckwheat, which had no pairs. Interestingly, the *SiABI5.3*, *SiABI5.4*, *SiABI5.12*, and *SiABI5.13* shared presence of homologous genes was observed in five additional species, excluding Tartary buckwheat, whereas *SiABI5.8* shared no homologous genes with any of the other six species (Table S5). These results suggest that *SiABI5.3*, *SiABI5.4*, *SiABI5.12*, and *SiABI5.13* may have existed before the differentiation of monocotyledons and dicotyledons and strongly influenced these species after differentiation, whereas *SiABI5.8* may not be evolutionarily homologous with the other six species. In general, *SiABI5* genes exhibited the highest degree of homology with maize, suggesting that these genes may have evolved from a shared ancestor present in diverse plant species. To clarify the evolutionary constraints acting on the *SiABI5* gene family, we calculated the Ka/Ks values of *SiABI5* gene pairs (Table S6). The Ka/Ks values for both subfamily-specific gene pairs of each and all segmental repeat gene pairs were less than one. This observation suggests that the *SiABI5* gene family was subjected to significant purification selection pressure throughout its evolutionary history.

### Evolutionary analysis of SiABI5 proteins and ABI5 genes of several other species

**Fig. 6:**
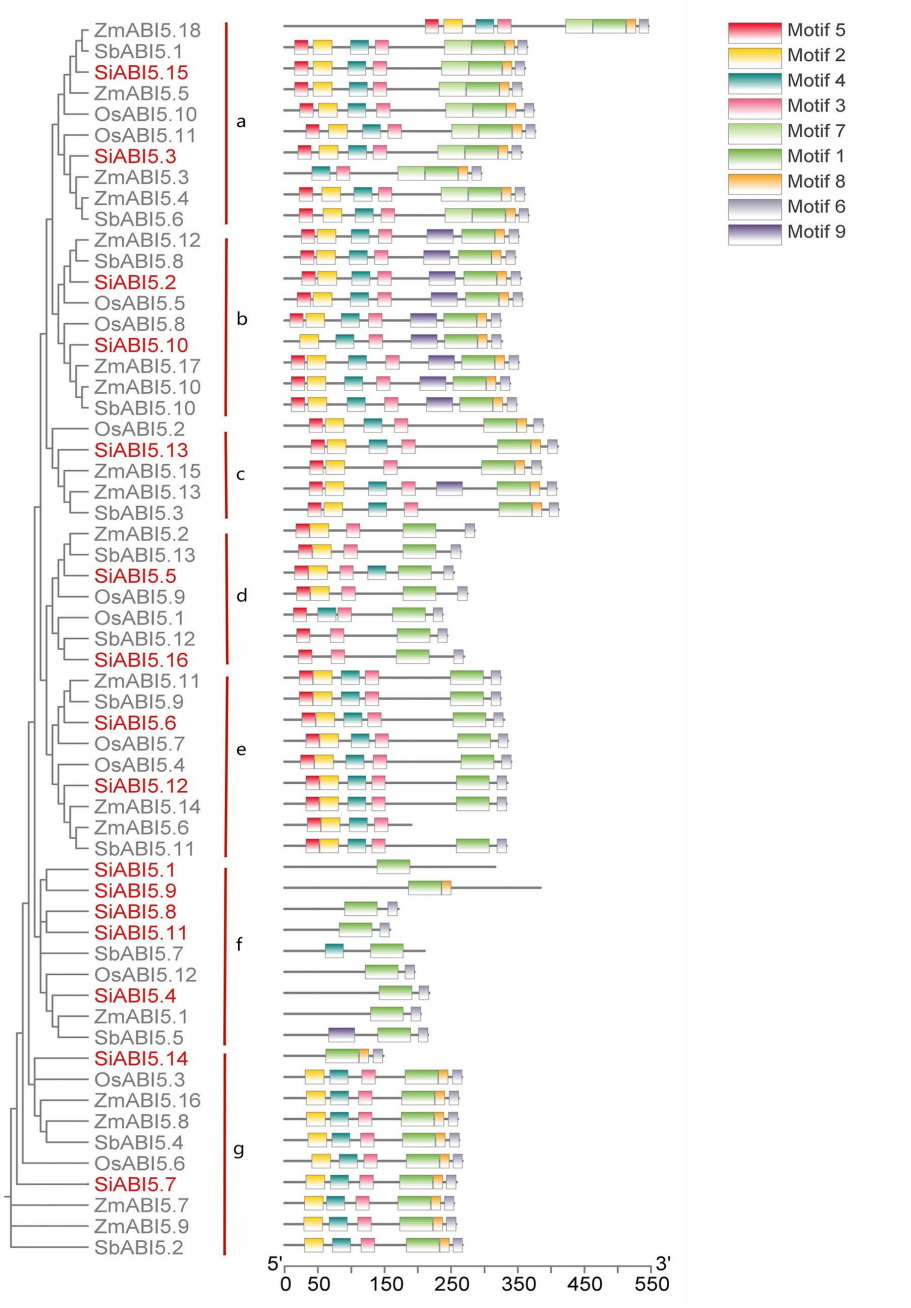
Phylogenetic relationships and motif compositions of ABI5 proteins from *S. italica* and three different plant species (*S. bicolor*, *O. sativa*, *Z. mays*). Evolutionary relationship between ABI5 proteins and motif composition. Different-colored boxes represent different motifs and their positions in each ABI5 protein sequence. Sequence information for each motif is provided in Table S2

We constructed phylogenetic trees of SiABI5 and ABI5 proteins from monocots using MEGA 11. Among them, the monocots included rice (12 proteins), sorghum (13 proteins), and corn (18 proteins) (Fig. 6, Table S7). As shown in Fig. 6, the ABI5 proteins can be divided into seven subfamilies in the phylogenetic tree (labeled a–g). Across the four species examined, each contributed to the presence of at least one *ABI5* gene within various subfamilies. Remarkably, the 16 *SiABI5* genes were distributed in a nearly equal manner among these seven subfamilies. Subgroup f had the most *SiABI5* genes (four), whereas subgroup c had the fewest *SiABI5* genes (one).

The analysis of conserved motifs within the SiABI5 protein was performed utilizing an online MEME analysis. Motif 1 exhibited a high degree of conservation and demonstrated an almost alternating distribution throughout the entire subfamily. These findings suggest that the composition of motifs is of critical importance for the *ABI5* genes and is potentially consequential for ABI5 proteins. However, relatively large differences existed between the different subfamilies. For example, “a” was the only subfamily to contain conserved motif 7. Subfamilies b, c, and f contained special motif 9, but only one gene in subfamilies c and f contained this motif. Subfamily b contained special motif 9 but lacked motif 7, and showed the opposite trend to that of subfamily a. In addition, subfamily e contained motif 5 but lacked motif 8. Subfamily f showed the greatest variation, containing motifs 1, 8, 9, and 4, but no other conserved motifs.

### Expression profiles of SiABI5 genes in various organs and at various developmental stages

**Fig. 7:**
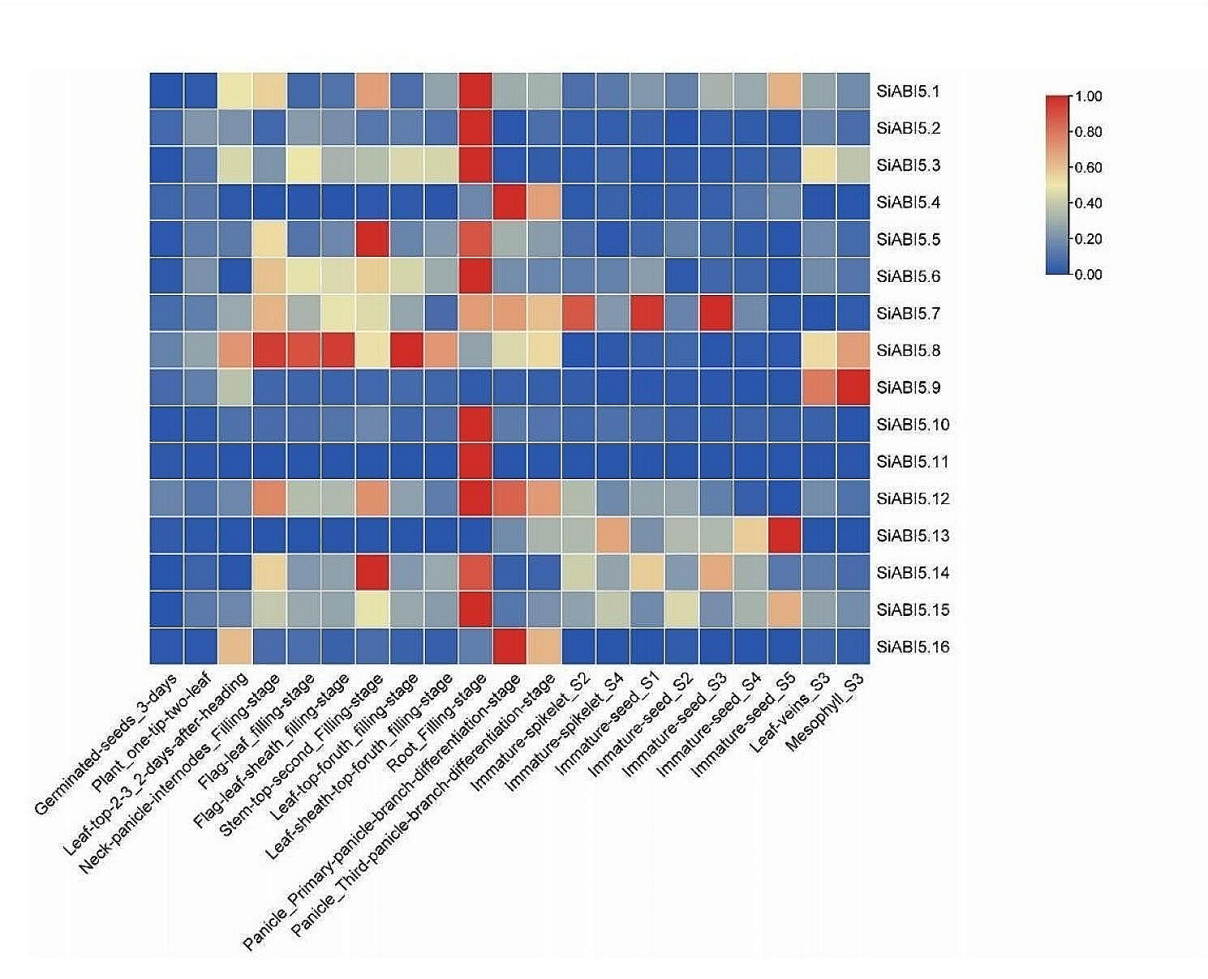
Relative expression patterns (FPKM value) of *ABI5*-group genes influencing various tissues and different developmental stages of *S. italica*. The color bar represents log2 expression levels (FPKM), and red and blue colors indicate high and low gene expression levels, respectively

We studied the expression profiles of *SiABI5* in roots, stems, leaves, panicles, and seeds at different developmental stages (Fig. 7). *SiABI5* exhibited the highest transcript levels in the roots, stems, and inflorescences of *S. italica*. Among them, the expression patterns of *SiABI5.1*, *SiABI5.2*, *SiABI5.5*, *SiABI5.6*, *SiABI5.10*, *SiABI5.11*, *SiABI5.12*, *SiABI5.14*, and *SiABI5.15* demonstrated a preference for roots. *SiABI5.7* and *SiABI5.8* were expressed specifically and abundantly in different tissues throughout the developmental process. Moreover, all genes showed very low expressions in the seeds at three days and at the two-leaf-one-spike stage of the plant. *SiABI5* expressions therefore differed between tissues, suggesting that some *SiABI5* genes are tissue specific. For example, the expression levels of *SiABI5.5* and *SiABI5.14* were higher in the stems than in other organs, whereas *SiABI5.4* and *SiABI5.16* showed a higher expression in inflorescences than in other organs. In addition, the expression patterns of the same genes varied by developmental stage.

### Expression pattern of SiABI5 genes during foxtail millet germination

**Fig. 8:**
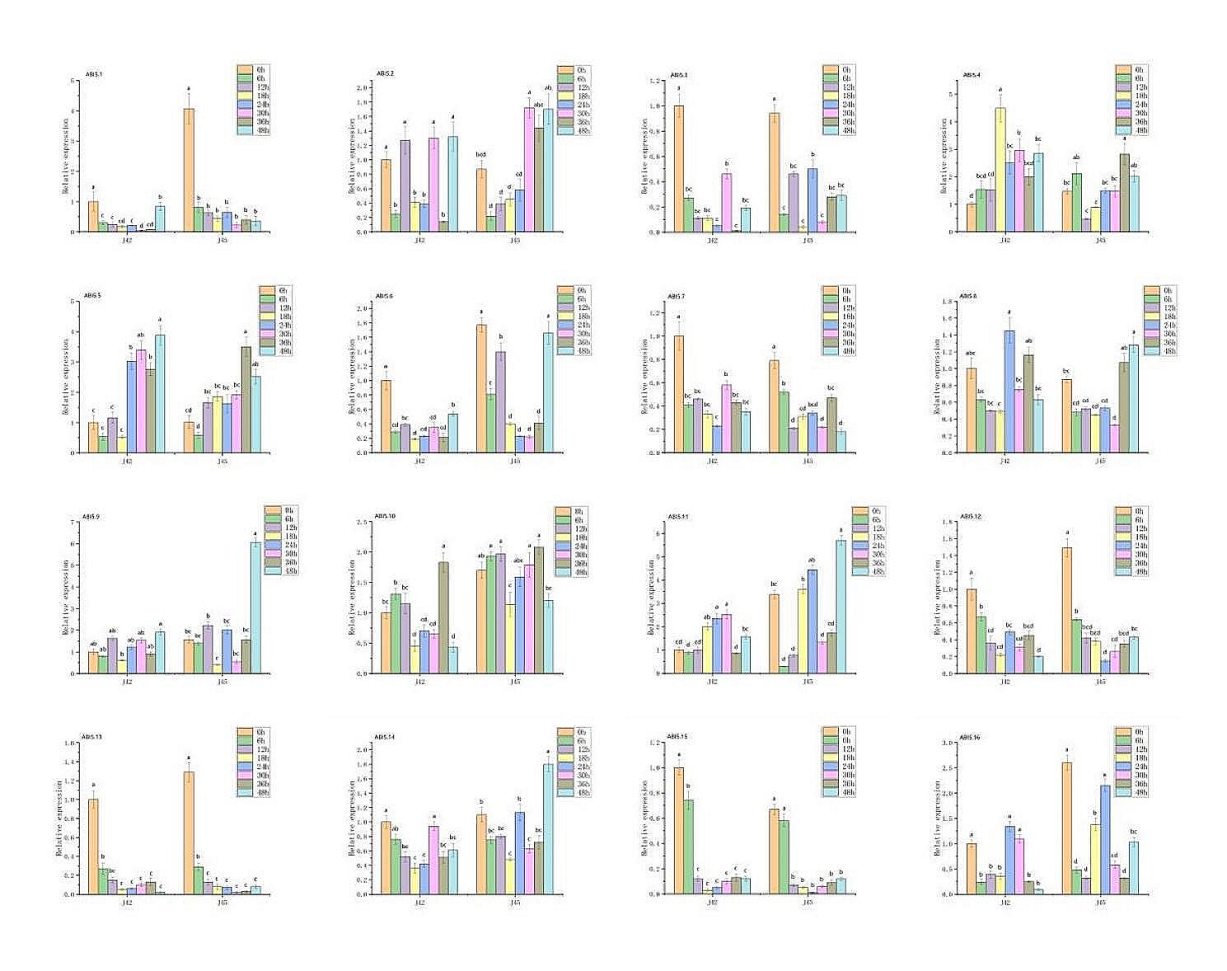
Expression patterns of 16 *SiABI5* genes at 0, 6, 12, 18, 24, 30, 36, and 48 h after germination determined using qPCR. Error bars were obtained from three measurements. Lowercase letters above the bar indicate significant differences (α = 0.05, LSD) between treatments

To more thoroughly characterize the *ABI5* gene function in *S. italica*, qPCR was used to determine the *SiABI5* gene expression in the Jingu 42 and Jingu 45 varieties at different times during germination. As shown in Fig. 8, *SiABI5.1*, *SiABI5.3*, *SiABI5.6*, *SiABI5.7*, *SiABI5.12*, *SiABI5.13*, *SiABI5.14*, *SiABI5.15*, and *SiABI5.16* were all highly expressed in the dried seeds. During the imbibition stage (0–6 h), the expression of 14 genes (excluding *SiABI5.4* and *SiABI5.10*) was downregulated. *SiABI5.4*, *SiABI5.5*, *SiABI5.9*, and *SiABI5.11* showed increasing expression rates during germination; therefore, these genes likely did not inhibit germination. Conversely, *SiABI5.1*, *SiABI5.3*, *SiABI5.6*, *SiABI5.7*, *SiABI5.12*, *SiABI5.13*, *SiABI5.15*, and *SiABI5.16* showed an approximate decreasing expression trend, indicating that these genes may inhibit germination. In addition, *SiABI5.5*, *SiABI5.6*, and *SiABI5.9* showed higher expression rates at 48 h after germination, indicating that these genes may play other roles during the seedling stage. In both varieties, the overall trends for most genes remained consistent from the early stages of germination to 48 h. Furthermore, four genes (*SiABI5.6*, *SiABI5.12*, *SiABI5.13*, and *SiABI5.15*) exhibited almost identical trends in both varieties.

### Expression patterns of SiABI5 genes during seed development in foxtail millet

**Fig. 9:**
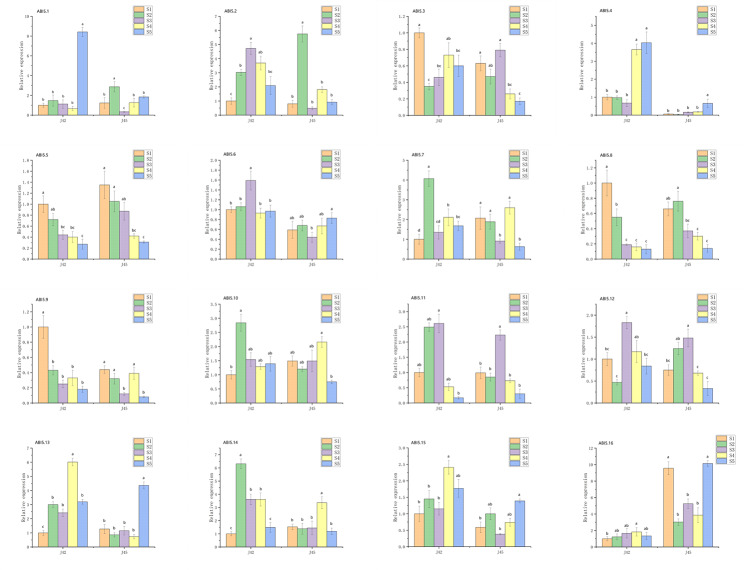
Expression patterns of 16 *SiABI5* genes during seed development stages S1–S5 determined using qPCR. Error bars were obtained from three measurements. Lowercase letters above the bar indicate significant differences (α = 0.05, LSD) between treatments

*ABI5* is an important regulator of seed maturation and is associated with seed storage products, desiccation tolerance, and seed longevity [31]. Therefore, we also investigated the expression of *SiABI5* during seed development (S1–S5). As shown in Fig. 9, *SiABI5.5*, *SiABI5.8*, and *SiABI5.9* exhibited a decreasing expression rate during grain maturation, with high expressions in the S1–S2 stages of seed development followed by a gradual decrease during seed maturation, which may have promoted early grain maturation. *SiABI5.4* showed an increasing expression during grain ripening and high expression at the S5 stage in both varieties, which may have promoted grain dehydration dormancy in later stages. *SiABI5.2*, *SiABI5.3*, *SiABI5.11*, *SiABI5.12*, and *SiABI5.14* initially exhibited increased expressions followed by decreases, suggesting that these genes may play a more prominent role during the middle stage of grain development and storage.

### 10. Expression patterns of SiABI5 genes in response to abiotic stresses

**Fig. 10:**
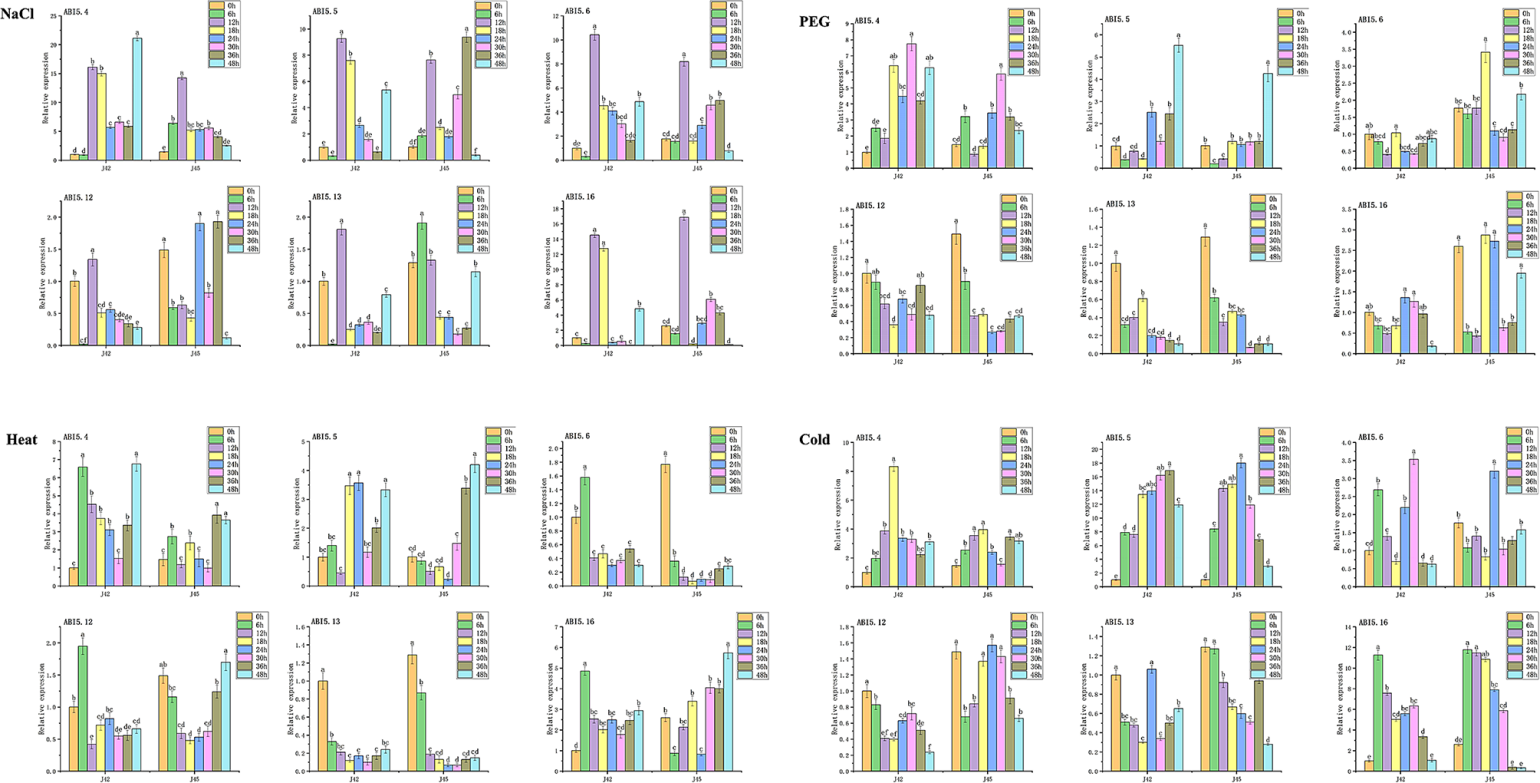
Expressions of six *SiABI5* genes subjected to abiotic stresses (salt, drought, heat, cold) at the seed germination stage (at 0, 6, 12, 18, 24, 30, 36, and 48 h) detected using qPCR. Error bars represent the standard error obtained from three measurements. Lowercase letters above the columns indicate significant differences between treatments (α = 0.05, LSD)

To further determine whether the *SiABI5* gene expression was affected by different abiotic stresses, six representative *SiABI5* members were selected for qPCR analysis during seed germination under different stress treatments (NaCl, drought, heat, and cold). Some genes were significantly expressed under stress conditions, whereas others were downregulated (Fig. 10). For example, under NaCl stress, all six *SiABI5* genes showed significantly elevated expression levels after 12 h, with *SiABI5.4*, *SiABI5.5*, *SiABI5.6*, and *SiABI5.16* particularly highly induced (more than five-fold). Moreover, the *SiABI5* genes in variety J42 showed decreased expressions following 6 h of salt stress, whereas those in variety J45 showed no clear change. Under drought stress, the expressions of all genes except *SiABI5.5* were higher than those under normal growth conditions. Compared to other stress conditions, heat stress had a minimal impact on the expression of *SiABI5* genes. Interestingly, upon heat treatment, the expression of *SiABI5.6* in J45 was lower than that under normal growth conditions, indicating that high temperatures promoted seed germination in certain aspects. Under the cold stress treatment, *SiABI5.5* and *SiABI5.16* were highly induced, with *SiABI5.16* showing a rapid response to stress and peaking at 6 h. In general, *SiABI5.4* and *SiABI5.16* exhibited higher levels of induced expression under stress conditions. Interestingly, the expressions of *SiABI5.12* and *SiABI5.13* increased in response to all stress conditions; however, these changes were relatively small, indicating relatively stable gene expressions.

### 11. Response of transgenic *Arabidopsis thaliana* overexpressing to osmotic stress

**Fig. 11:**
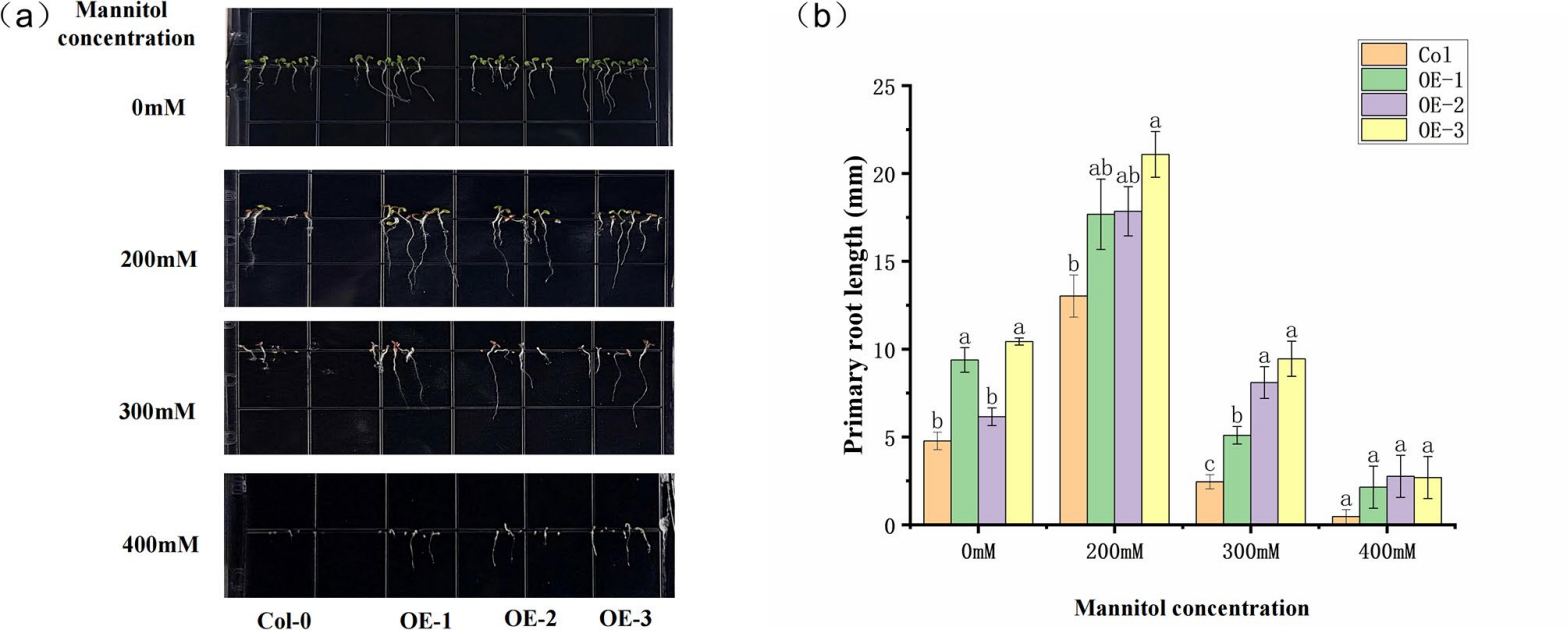
Osmotic stress analysis of *SiABI5.12* overexpression plants. (**a**) One-week-old WT and *SiABI5.12* overexpression lines under 0, 200, 300, 400 m*M* mannitol. (**b**) Primary root length of seedlings after 7 days mannitol stress. Error bars represent the standard error obtained from six measurements. Lowercase letters above the columns indicate significant differences between genotypes (α = 0.05, LSD)

To explore the significance of *SiABI5* during stress response, *SiABI5.12* was ectopic expression in *Arabidopsis* and three transgenic lines were used to detect the response to osmotic stress (Fig. 11). Under normal conditions, no obvious phenotypic alterations were observed among WT and transgenic lines. With the increase of mannitol concentration, the germination rate decreased in WT. In contrast, the germination rate of overexpressed lines was higher than that of WT under osmotic stress. Transgenic lines were more tolerant to osmotic stress and had a higher percentage of green cotyledons compared to the wild type under 200 mM mannitol (Fig. 11a). At the same time, regardless of mannitol concentration, overexpressed *Arabidopsis* had a significant advantage in primary root length. No obvious alterations in the root length of OE-2, OE-3 lines were observed between 300 mM and 0 mM mannitol, while significant decrease in wild-type. These results indicating that ectopic expression of *SiABI5.12* in Arabidopsis greatly increases the tolerance to osmotic stress at gemination stage.

## Discussion

### Identification and analysis of SiABI5 genes in foxtail millet

ABI5 is the key transcriptional regulator of ABA-dependent gene expression whose members crucially influence plant development and adaptation to adverse environmental conditions [32–35]. Specifically, the *ABI5* gene is highly integral in regulating seed maturation, germination, and flowering time, as well as the responses to drought, salt, and high/low-temperature stress [36]. The advancement of metagenomics in recent years has led to the discovery and characterization of *ABI5* genes in various plant species, such as *Arabidopsis thaliana, Z. mays, S. tuberosum*, and *O. sativa*. However, no previous studies have reported the role of the *SiABI5* gene in the new C4 model crop, foxtail millet.

In this study, we identified 16 *SiABI5* members. The physicochemical analysis of these 16 *SiABI5* genes showed a relative molecular weight range of 22.4–43.3 kDa, with an isoelectric point range of 5.32–9.97. According to the subcellular localization of ABI5 proteins, most SiABI5 proteins were present in the nucleus and chloroplasts. Due to the evolutionary conservation of subcellular localization, the presence of homology to a protein with known localization is often a useful indicator of actual protein localization [37]. In addition, all 16 SiABI5 transcription factors had highly conserved bZIP domains, which play significant roles in regulating various biological processes [38]. Genes belonging to the same group of bZIP genes exhibit identical or similar functions, thereby serving as a valuable reference for investigating the functional characteristics of this gene family. The comparative genomic analysis of the gene structure revealed similar intron–exon structures within the same subfamily, as well as substantial differences between different subfamilies, which allowed us to speculate that these genes appeared to share a common evolutionary origin and molecular functions. Introns, within the context of plant evolution, have the potential to both increase gene length and enhance the frequency of recombination between genes, thus demonstrating an integral regulatory function [39].

Gene promoters refer to DNA sequences situated upstream of gene-coding regions encompassing numerous cis-acting elements that serve as specific binding sites for proteins responsible for initiating and regulating transcription processes [40]. The regulation of gene expression by cis elements in the promoter region has become the main mechanism by which organisms adapt to different environments [41]. In the present study, various cis-acting elements were identified in 16 *SiABI5* genes. The presence of light-response-related elements in the majority of *SiABI5* genes indicated the significant involvement of these elements in the light response of plants. Many cis-acting elements, including MeJA, ABA, SA, GA, and auxin, influenced the response to hormones and biotic stresses. The components of the auxin, cytokinin, abscisic acid, jasmonic acid, and brassin steroid signaling and metabolic pathways either helped regulate *ABI5* or are regulated by *ABI5* [6]. In addition, many *SiABI5* genes possessed elements related to abiotic stress, such as wounds, cold, heat, anaerobic induction, and drought. This also indicates that *SiABI5s* play an important regulatory role under external stress. In conjunction with prior research findings, our analysis suggests that *SiABI5* genes participate in the transcriptional regulation of both plant growth and stress responses [28, 42].

### Evolution of SiABI5 genes

Most repeat events in the foxtail millet genome are generated during whole-genome duplication events shared by the Poaceae family [43]. In this study, 16 *SiABI5* genes were identified in foxtail millet (diploid). Because gene duplication is a major driving force of gene amplification and evolution, duplicate events were also observed in the aforementioned plants. Four and eleven gene duplication events were found in *Arabidopsis* and tomato plants, respectively, whereas no gene duplication events were found in buckwheat. Among monocot plants, 45, 38 and 37 collinear gene pairs of foxtail millet *SiABI5* gene were found in maize, rice and sorghum, respectively. Most of the *ABI5* gene duplication events mentioned above were segmental duplications. These results suggest that differences in the number of *ABI5* genes between dicots and monocots may be caused by gene duplication. Finally, to more fully illustrate the evolutionary limitations affecting the *SiABI5* gene family, non-synonymous and synonymous substitutions in *SiABI5* were analyzed. The findings demonstrate that the Ka/Ks ratios of both gene pairs and segmental duplicate gene pairs within each subfamily were consistently below one. This indicates that the *SiABI5* gene family has undergone significant purification and has been subject to rigorous selection pressures throughout its evolutionary trajectory.

### Spatio-temporal expression patterns of SiABI5 genes and responses to abiotic stress

Genetic studies have shown that *ABI5*, together with other genes, may influence ABA signal transduction as an important regulator of seed maturation and germination [28]. Several transcription factors regulate the expression of ABI5 during the germination of *Arabidopsis* seeds [18, 44–46]. For example, the transcription factors ABI3, ABI4, AGL21, Myb7, and RAV1 are implicated in the regulation of seed germination by ABA, primarily via their regulatory control over the transcription factor ABI5 [18, 44–46]. During the seedling stage, the *ABI5* expression has been detected in root tips, nodes, and leaf veins, as well as at the edges of leaves and flowers in older plants [18]. *OsABI5* is highly expressed in mature pollen, suggesting that it may strongly influence pollen maturation [47]. In our study, some *ABI5* members were extensively expressed in foxtail millet tissues, such as *SiABI5.7* and *SiABI5.8*. The expression levels of *SiABI5.5* and *SiABI5.14* were higher in the stems than in other organs, whereas the *SiABI5.4* and *SiABI5.16* expressions were higher in the inflorescences than in other organs. Each gene appears to provide integral functions in different tissues and species. We also identified subtle differences in these structures among the different tissues, which clarified how transcription factors regulate tissue-specific biological processes. During germination, the expression levels of *SiABI5.1*, *SiABI5.3*, *SiABI5.6*, *SiABI5.7*, *SiABI5.12*, *SiABI5.13*, *SiABI5.15*, and *SiABI5.16* decreased, which may indicate their role in germination inhibition. Moreover, *ABI5* regulates seed maturation and longevity in legumes [31]. In our study, most genes were highly expressed in the middle stage of grain development, indicating that *SiABI5* may crucially influence this stage of grain maturation.

*ABI5* participates in ABA networks and is activated in response to abiotic stress to regulate the expression of many genes and influence plant resilience. For example, *DELLA* modulates the stability of *ABI5*. Within the ABA signaling pathway, the stability of *ABI5* is influenced by ABA receptors (PYLs); moreover, *ABI5* actively regulates the expression of *PYLs* in a positive manner [48]. *AtABI5* serves as a central hub for the crosstalk between plant hormones. The *ABI5* expression is also regulated at the protein level, interacting with numerous proteins by regulating their stability or activity as transcription factors [49, 50]. Increasing evidence suggests that *ABI5* serves as a key inhibitor of multiple signaling pathways involved in seed germination regulation. These pathways include those mediated by ABA, GA, light signals, auxin, BR, JA, and other signaling molecules [51]. Because *ABI5* integrally impacts plant resistance to environmental stresses, its expression is often induced when plants encounter abiotic stress. For example, *OsABI5* expression is upregulated by ABA and high salinity, but downregulated by drought and cold (below 4 ℃) in seedlings [28]. *ZmABI5* is strongly induced by ABA treatment and by salinity, high-temperature, and wounding stresses [25]. Arabidopsis *ABl5* can be combined with the *cis-*element ABRE to regulate the expression of a large number of downstream drought resistance, saline-alkali tolerance and other related functional genes, affecting plant stress resistance. In this study, six stress-related *SiABI5* genes all had cis-acting elements involved in abscisic acid reaction ABRE (ACGTG). The cis-acting element associated with low temperature LTR (CCGAAA) were predicted in *SiABI5.5* and *SiABI5.16*, both of which showed high expression levels under the cold treatment (Fig. 10). cold and NaCl stress had the greatest impact, and more genes tended to reach their highest expression levels during the early stages of germination. At the same time, *SiABI5.4* and *SiABI5.16* exhibited greater upregulation under stress conditions, implying a multifaceted role in abiotic stress responses. Although drought -inducible associated elements MBS (CAACTG) were not predicted in *SiABI5.12* and *SiABI5.16*, their expression was also increased under PEG treatment. *ABI5.12* responded to the germination, seed development, and abiotic stress. These results are basically consistent with the prediction analysis of cis-acting elements of promoters. In addition, ectopic expression *of SiABI5.12* in Arabidopsis greatly increases the tolerance to osmotic stress at gemination stage (Fig. 11), which demonstrated the function of SiABI5 responded to abiotic stress. The mode of the *ABI5* gene and ABI5 protein regulation is complex and requires many transcription factors, enzymes and protein regulators. *ABI5* expression is the outcome of the action of many transcription factors such as WRKY and MYB as well as epigenetic events [18]. Thus, further investigation of the biological functions of these genes may provide strategies for enhancing millet yields under various abiotic stress conditions.

## Conclusions

In this study, we summarized the essential characteristics of the *SiABI5* family of foxtail millet, including its phylogeny, physical and chemical properties of proteins, gene structure, chromosomal localization, duplicated events, cis-acting elements, and gene expression patterns. The identification and systematic analysis of *ABI5* genes in foxtail millet revealed 16 *SiABI5* genes that were unevenly distributed on seven chromosomes and divided into five clades. An analysis of gene duplication events indicated that several segmental and tandem duplications occurred during the expansion of the *SiABI5* family. Expression profile analyses of the *SiABI5* genes showed that most *SiABI5* genes were highly expressed in the roots. qPCR results for the *SiABI5* gene at different germination and maturation stages showed that the *SiABI5* gene was highly expressed in dry seeds and during the middle stage of seed maturation. The expressions of six *SiABI5* genes were analyzed under stress conditions; most *SiABI5* genes were upregulated under abiotic stress in foxtail millet, which confirmed that the *SiABI5* family widely influenced the abiotic stress response. Overexpression of *SiABI5.12* in Arabidopsis enhance resistance to osmotic stress. These results help clarify the response of *SiABI5* to abiotic stress and lay a foundation for further functional research on *ABI5* in foxtail millet crops.

## Materials and methods

### SiABI5 gene identification, phylogenetic analysis, and physicochemical properties

To identify candidate *SiABI5* genes, we utilized the *Arabidopsis ABI5* gene (https://www.Arabidopsis.org/) and the rice *ABI5* gene (http://Rice.plantbiology.msu.edu/index.shtml) as references. By performing a genome-wide blast analysis on the foxtail millet sequence (http://foxtail-millet.biocloud.net/home), we obtained potential *SiABI5* genes using thresholds of a score value of ≥ 100 and an e-value of ≤ 1e − 10. Then, we downloaded the hidden Markov model of the bZIP domain (PF00170) in the Pfam database (http://pfam.xfam.org/) and searched for ABI5 proteins using the HMMER 3.0 software (default parameters) (http://HMMER.org/). All candidate *SiABI5* genes were verified using the SMART software (http://smart.embl.de/). We constructed a phylogenetic tree using the MEGA 11 software (Mega Limited, Auckland, New Zealand) and employed the neighbor-joining method with a bootstrap value of 1,000. Finally, 16 *SiABI5* genes were obtained, which were analyzed according to their protein length, molecular weight, isoelectric point (https://web.expasy.org/compute_pi/), and protein subcellular localization prediction (https://wolfpsort.hgc.jp/).

### Gene structure, motif compositions, gene synteny, and promoter analysis of SiABI5 genes

The physical location of the *SiABI5* gene was derived from the Yugu genome, and, subsequently, mapping to chromosomes was performed. Gene duplication events in the *SiABI5* genes were analyzed for collinearity using the Multiple Collinearity Scan toolkit X (MCScanX) employing default parameters. Furthermore, a dual-system plot was employed to assess the homology of *SiABI5* genes across different species. Phylogenetic trees were constructed using protein sequences (*Oryza sativa*, *Sorghum bicolor*, and maize) downloaded from the Phytozome (https://phytozome-next.jgi.doe.gov/) database using Muscle Wrappers. To construct a gene structure diagram for *SiABI5*, the coding sequence was compared with the corresponding genomic DNA sequence. Additionally, TBtools was employed to compile an intron–exon map based on the genome annotation information of *S. italica*. A protein motif analysis was performed using the MEME database (http://meme-suite.org/tools/meme). We extracted the promoter sequence 2,000 bp upstream of the *SiABI5* gene and analyzed the cis-acting elements of the promoter region in PlantCARE (http://bioinformatics.psb.ugent.be/webtools/plantcare/html/).

### Expression profiles of SiABI5 genes

The FPKM values of *SiABI5* genes in various tissues, including roots, stems, and leaves, were obtained from the Multi-omics Database for *S. italica* (http://foxtail-millet.biocloud.net/home). The TBtools software was employed to create expression heatmaps for the *SiABI5* genes.

### Plant material

Two foxtail millet varieties Jingu 42 (J42) and Jingu 45 (J45), which were developed and provided by Shanxi Agricultural University, were used in this experiment. Seeds were sterilized in 75% ethanol (v/v) for 5 min and rinsed with distilled water. Then, the seeds were germinated in a box at a temperature of 26 °C and relative humidity of 65% in a dark chamber incubator. In addition, the germinated foxtail millet plants were subjected to four abiotic stress conditions (salt: 150 mM NaCl, drought: 18% PEG6000; heat: 35 ℃, cold: 15 ℃). For the salt and drought stressors, different replicates were immersed in the same volume of liquid under the same stress conditions. Samples were collected at 0, 6, 12, 18, 24, 30, 36, and 48 h for subsequent qPCR analysis. The expression levels of six representative *SiABI5* genes were also determined. For the experimental materials grown in the field, we obtained grains at filling stages S1–S5. In the initial grain-filling stage (S1), the grains displayed a green coloration and contained water emulsions. In the middle stage of grain-filling (S2), the grains maintained their green hue and continued to possess water emulsions, and concurrently underwent endosperm formation. In the late stage of grain-filling (S3), the grains exhibited a yellow-green coloration and the internal contents progressively solidified, allowing for the separation of the endosperm from the grain contents. In the final stage of grain-filling (S4), the grains exhibited a yellow hue and their internal contents solidified. Moreover, the endosperm could not be separated from the grain contents. In the stage of grain maturation (S5), the grains were dark yellow, hard, brittle, and mature. Samples were collected from plants grown under identical conditions (five replicates), then immediately frozen in liquid nitrogen and stored at ‒80 °C for future use. The expression levels of the 16 *SiABI5* genes were then determined.

### RNA isolation and qPCR

RNA was isolated from the leaves using a TRIzol kit (Accurate Biotechnology, Changsha, China). cDNA was synthesized using a reverse transcription kit (Accurate Biotechnology, Changsha, China) and real-time PCR was performed using the SYBR Green dye method (Accurate Biotechnology, Changsha, China). The PCR primers were designed using Primer Premier 5 software (Vancouver, BC, Canada) and are listed in Table S8. The qPCR reaction was accomplished in a Bio-Rad CFX system, with a 10 µL reaction system containing 5 µL of TB Green premix Ex Taq II, 1 µL of cDNA, 0.5 µL each of forward and reverse primers, and 3 µL of RNase-free water. The qPCR reaction conditions were set according to the manufacturer’s instructions. SiActin was used as an internal standard, and the expression levels of each gene were calculated using the 2^−ΔΔCt^ method.

### Analysis of transgenic plants resistance to osmotic stress

*Arabidopsis* seeds were sterilized in 75% ethanol (v/v) for 5 min and rinsed with distilled water. Placed in 1/2 MS petri dishes containing mannitol (concentrations of 0, 200, 300 and 400 mM, respectively), sealed and placed in a constant temperature culture chamber. The root length of 7-day *Arabidopsis* seedlings was measured with a ruler.

### Statistical analysis

The SPSS 26 software was employed to conduct a one-way analysis of variance (ANOVA) on the data, followed by a comparison with the least significant difference (LSD) at significance levels of 0.05 and 0.01. Subsequently, bar charts were drawn using the OriginPro2022b software.

### Electronic supplementary material

Below is the link to the electronic supplementary material.

Supplementary Material 1. Physical and chemical properties of 16 *SiABI5* genes

Supplementary Material 2. Motif Composition

Supplementary Material 3. Cis-regulatory elements

Supplementary Material 4. Intraspecific collinearity

Supplementary Material 5. Interspecies collinearity

Supplementary Material 6. Ka/Ks ratio

Supplementary Material 7. ABI5 protein sequence information

Supplementary Material 8. Primer information

## Data Availability

The whole genome sequence information of foxtail millet was obtained from the Home genome website (https://foxtail-millet.biocloud.net/home). In the experiment, the foxtail millet material used was provided by Shanxi Agricultural University. The datasets supporting the conclusions of this study are included in the article and its additional files.
